# Electrochemical Synthesis of Mesoporous CoPt Nanowires for Methanol Oxidation

**DOI:** 10.3390/nano4020189

**Published:** 2014-03-28

**Authors:** Albert Serrà, Manuel Montiel, Elvira Gómez, Elisa Vallés

**Affiliations:** Physical Chemistry Department and Institute of Nanoscience and Nanotechnology (IN2UB), University of Barcelona, Martí i Franquès 1, 08028 Barcelona, Spain; E-Mails: a.serra@ub.edu (A.S.); manuel.montiel@ub.edu (M.M.); e.gomez@ub.edu (E.G.)

**Keywords:** mesoporous nanowires, CoPt alloy, electrodeposition, microemulsion, ionic liquid DMFC

## Abstract

A new electrochemical method to synthesize mesoporous nanowires of alloys has been developed. Electrochemical deposition in ionic liquid-in-water (IL/W) microemulsion has been successful to grow mesoporous CoPt nanowires in the interior of polycarbonate membranes. The viscosity of the medium was high, but it did not avoid the entrance of the microemulsion in the interior of the membrane’s channels. The structure of the IL/W microemulsions, with droplets of ionic liquid (4 nm average diameter) dispersed in CoPt aqueous solution, defined the structure of the nanowires, with pores of a few nanometers, because CoPt alloy deposited only from the aqueous component of the microemulsion. The electrodeposition in IL/W microemulsion allows obtaining mesoporous structures in which the small pores must correspond to the size of the droplets of the electrolytic aqueous component of the microemulsion. The IL main phase is like a template for the confined electrodeposition. The comparison of the electrocatalytic behaviours towards methanol oxidation of mesoporous and compact CoPt nanowires of the same composition, demonstrated the porosity of the material. For the same material mass, the CoPt mesoporous nanowires present a surface area 16 times greater than compact ones, and comparable to that observed for commercial carbon-supported platinum nanoparticles.

## 1. Introduction

More than 160 years ago, the conversion of chemical energy into electrical energy in a primitive fuel cell was demonstrated as an attractive technology due to the significant possible environmental benefits and system efficiencies [1]. However, fuel-cell systems have proved difficult to develop viable industrial products, due to the material or manufacturing cost [2,3]. Nowadays, the needs of modern society and the emerging ecological claims show an unquestionable interest in low-cost, scalable, effective, and environmentally friendly energy conversion and storage devices [4,5,6,7]. Therefore, these characteristics depend intimately on the properties of the constituent materials. In the last decades, the use of nanomaterials has been an emergent topic due to the unusual properties (mechanical, electrical, optical, among others) and the high surface-volume ratio of these materials [8,9,10,11]. It provides an enormous challenge to combine the advantages and disadvantages of nanomaterials in energy conversion and storage devices, especially to take advantage of the high specific area of them in catalytic routes to convert fuels into energy [12,13].

Among the various categories of fuel cells, Direct Alcohol Fuel Cells (DAFCs) working at low temperatures (<373 K) and employing proton exchange membranes, are promising devices for electrochemical power generation [14,15]. Especially, methanol (DMFCs) or ethanol (DEFCs) fuel cells are attractive systems to supply energy to portable electronic devices, due to the high energy density and conversion efficiency, fuel availability, low-to-zero pollutant emission, and because they can be easily handled [16,17,18]. However, the slow oxidation kinetics of these fuels inhibits the wide use of these systems due to the need to use noble metal derivatives as catalysts (Pt, Pd, *etc.*). In order to resolve these critical problems, more effective and inexpensive materials should be synthesized, which enhance the kinetics of alcohol oxidation and the activity for oxygen reduction. Recently, the use of nanoparticulated bimetallic platinum alloys with less expensive 3d-transtion metals like Fe or Co, among others, promotes the electrocatalytic activity and reduces costs [19,20].

Electrodeposition technique has been shown a useful tool for preparation of nanostructured materials over several conductive substrates, even over templates, photolithographically prepared substrates orin soft-template systems (microemulsions and self-assembled monolayers) [21,22,23,24]. Microemulsions are liquid systems of water, oil and surfactant, which are optically transparent and thermodynamically stable. In recent years, classical microemulsions have been widely used to chemically synthesize nanoparticles in water or oil droplets of a few nanometers stabilized in a continuous oil or water, respectively [25,26,27]. Recently, in our laboratory, we have demonstrated the possibility to use microemulsions based on ionic liquids due to their intrinsic ionic conductivity, low vapor pressure and wide electrochemical window [28]. These systems provide more conductivity and lower ohmic resistance than classical microemulsions, which use oil component (dielectric component).

Mesoporous nanomaterials could be prepared by several different routes including phase separation, controlled foaming, among other strategies [29,30]. Synthesis and applications of mesoporous materials, especially ordered mesoporous, have received intensive attention because of their large surface areas, uniform pore size, and tunable periodic morphologies. These materials are promising candidates as nanoreactors, catalysers, sensors or drug deliverers [31,32,33].

The aim of this work is the preparation, by means of electrodeposition method, of highly porous CoPt nanowires, catalytic to methanol oxidation. In order to electrodeposit directly the mesoporous nanowires, an ionic liquid in water microemulsion (IL/W microemulsion) was selected. The solubility of the salts in the ionic liquid must be very low respect to that in water to avoid the electrochemical deposition from the IL. Thus, an electrolytic solution containing the platinum and cobalt salts was used as aqueous component, and 1-Butyl-3-methylimidazolium hexafluorophosphate (bmimPF_6_) as ionic liquid distributed as droplets into the aqueous solution. The objective is to replicate the structure of the IL/W microemulsion to the CoPt nanowires grown in it. CoPt should deposit in the interior of the membrane pores, only from the aqueous component, giving as a results a mesoporous structure of the nanowires. This proposal supposes a totally new methodology for preparing mesoporous nanowires as the use of microemulsions had never proposed previously. However, both mesoporous nanorods and mesoporous films have been prepared using aqueous surfactant solutions (with very low surfactant content) [34,35,36,37]. Therefore, our methodology introduces a new strategy, which would seem useful to prepare other materials like mesoporous polymeric nanowires, according to the more robust template capability of microemulsion than micelle aqueous solution.

## 2. Results and Discussion

**Figure 1:**
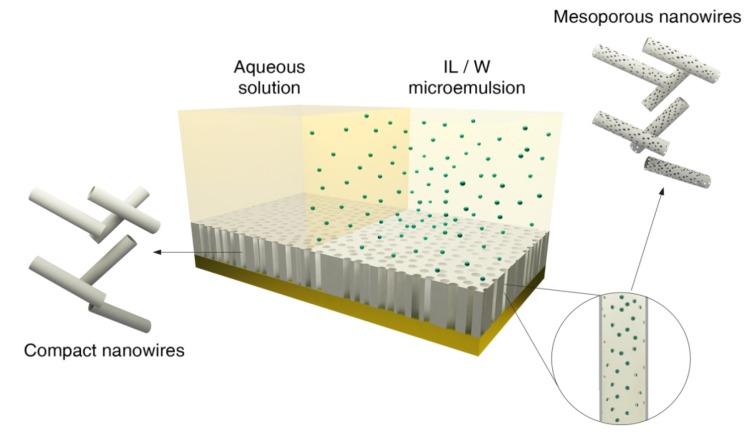
Schematic representation of electrochemical synthesis of mesoporous and non-mesoporous CoPt nanowires on polycarbonate membranes coated with gold layer.

In order to synthesize mesoporous nanowires of CoPt alloy to enhance the catalytic activity to methanol oxidation, an ionic liquid-in-water (IL/W) microemulsion has been considered as a template to control the pore size (Figure 1). Several studies have demonstrated a low solubility of electrolytic species in this ionic liquid [28]. This allow us to propose a new interesting electrochemical route to obtain different nanostructured materials, in this case mesoporous materials, as a consequence of the non-deposition of the material from the ionic liquid of the microemulsion. We select an IL/W microemulsion based on literature [38] with 77.1 wt.% of aqueous solution (W), 20.7 wt.% of Triton X-100 (S) and 2.2 wt.% of bmimPF_6_ to test if it is useful for the electrochemical synthesis of the CoPt mesoporous nanowires. In order to synthesize electrochemically mesoporous nanowires we will combine the use of polycarbonate membranes and microemulsion soft-templates. However, we must consider different properties of the selected microemulsion that can affect the possibility to follow this synthesis route, such as surface tension, conductivity, viscosity and hydrodynamic radius of microemulsion droplets. The surface tension can affect the wetting of the membrane’s channels, the conductivity of the microemulsion can condition the deposition rate, a high viscosity of the microemulsion could difficult the filling of the membrane’s pores and the hydrodynamic radius of microemulsion droplets define the pore size and distribution. We have determined the values of theses magnitudes and Table 1 shows the surface tension, relative density, viscosity and conductivity of both aqueous solution and microemulsion and the hydrodynamic radius of microemulsion droplets. Surface tension of the IL/W microemulsion decreases respect to that of CoPt aqueous solution, which can favour the wetting of the channels walls of the membranes, but the higher value of the viscosity for the microemulsion allows us expecting lower deposition rate of the CoPt nanowires than in aqueous solution. IL/W microemulsion presents lower conductivity than aqueous medium due to the intrinsic nature of each system, but due to that the aqueous solution is the continuous component of the microemulsion, conductivity is not drastically different for both systems. For this reason, a low influence of the conductivity on the electrodeposition process is expected. Therefore, the major effect in electrodeposition process in polycarbonate membranes should be the transport through the channels. In order to measure the hydrodynamic diameter of the droplets and the polydispersity index (PDI) by Dynamic Light Scattering (DLS), the refraction index of microemulsion system was determined (*n*_IL/W_ = 1.3598). DLS measures leads to 4.2 nm of hydrodynamic diameter of ionic liquid droplets and 0.1 of polydispersity index (PDI), which permits to expect a uniform pore size distribution according to the monodisperse droplets size distribution. A value of 12.8 nm has been determined in the literature for a similar microemulsion but containing pure-water [38], which shows that the presence of the cobalt and platinum salts in the aqueous component influences, as expected, the microemulsion characteristics.

**Table 1:** Surface tension (γ), viscosity (η), relative density (δ_r,H_2_O_), conductivity (ҡ), and hydrodynamic diameter (*D*_h_) of aqueous solution (W) and ionic liquid in water microemulsion (IL/W microemulsion) at 25 °C.

Properties	Aqueous solution (W)	IL/W microemulsion
γ (mN·m^−1^)	71.9	18.8
η (mPa·s)	0.84	22.3
δ_r,H_2_O_	1.00	1.02
ҡ (mS·cm^−1^)	11.18	8.42
*D*_h_ (nm)	-	4.20 ± 0.08

In all cases, in order to favor the initial filling of the membrane’s channels for the CoPt deposition, membranes were introduced in the aqueous solution or in the microemulsion for long time (24 h). After this, the electrochemical synthesis of the CoPt nanowires was performed in the IL/W microemulsion and the resulting nanowires were compared with those obtained in aqueous solution. Previously, an electrochemical study of the process in each system was necessary to define the potentials adequate to obtain the CoPt nanowires. As gold seed layers were used to make conductive the membranes, the electrochemical study of the deposition process was initially performed on Si/Ti (15 nm)/Au (100 nm) substrates and after on polycarbonate membranes/Au (100 nm) substrates to detect possible influence of the porous template.

**Figure 2:**
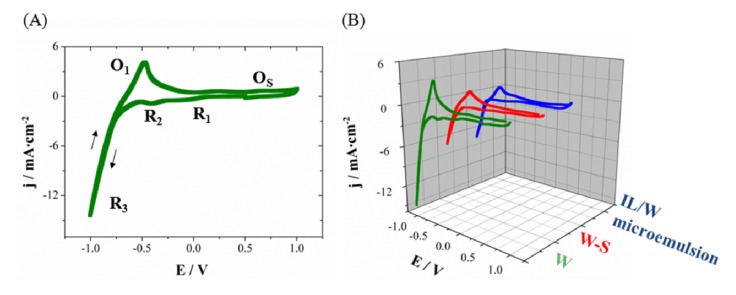
Cyclic voltammetry at 25 °C and stationary conditions on Si/Ti (15 nm)/Au (100 nm) of (**A**) CoPt solution (W) and (**B**) CoPt solution (W), aqueous solution–surfactant system (79W:21S) and IL/W microemulsion.

From the voltammetric results on Si/Ti/Au substrates, the deposition process of CoPt system in aqueous solution (W), aqueous solution-surfactant (79W:21S) or IL/W microemulsions systems seem occur in a similar way. Figure 2 shows the cyclic voltammogram of each system at 50 mV·s^−1^. The selected cathodic limit (−1.0 V) allows detecting the main reduction processes: platinum (IV) reduction (*R*_1_), protons reduction over the first deposited platinum (*R*_2_) and simultaneously reduction of cobalt and hydrogen evolution (*R*_3_). In the anodic scan, the oxidation of retained molecular hydrogen over the surface electrode (*O*_1_) and the quasi-simultaneously surface oxidation of platinum and gold (*O*_S_) were detected. The oxidation of the CoPt is difficult to detect as corresponds to a noble platinum alloy [39]. The voltammetric study allows detecting the same processes from IL/W microemulsion, but its major viscosity and slightly lower conductivity implies lower current densities of the processes, *i.e.*, lower deposition rate. Intermediate current densities were observed for the aqueous solution-surfactant system; this solution was studied to analyze the surfactant effect and to determine the veritable effect of microemulsion in nanowire characteristics.

From the voltammetric results, a potential of −1000 mV was selected to perform the CoPt codeposition, firstly on the Si/Ti/Au substrates, from aqueous solution (without or with surfactant) and IL/W microemulsion. Deposits were prepared at 25 °C without stirring. At the selected potential, CoPt films of similar composition were obtained in all cases (Table 1), which demonstrated that the nature of each system, continuous or discrete media does not affect the deposition process. Metallic appearance films were obtained from both W and W-S systems, whereas black ones are obtained from the IL/W microemulsion, which reveals the major roughness of the films. The measure of the thickness of the CoPt deposits permits calculating the efficiency of the electrodeposition process. The efficiency was estimated by comparing the calculated and experimental charge densities by means the following equation:

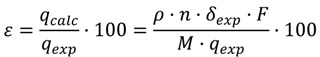
(1)
where *q*_calc_ is the calculated charge density corresponding to the deposit formation, *q_exp_* is the experimental circulated charge density, ρ is the Co*_x_*Pt_1__−*x*_ density estimated from the composition and crystalline cell volume, *n* is the total number of electrons, *V*_calc_ is the deposit volume, *F* is the Faraday constant, and *M* is the molecular weight of Co*_x_*Pt_1__−*x*_.

From these results, CoPt electrodeposition is possible from the three studied systems, leading to films of similar composition but different efficiency of the process. In all cases, low values of efficiency were obtained due to the catalytic behavior of platinum alloys to hydrogen evolution.

**Figure 3:**
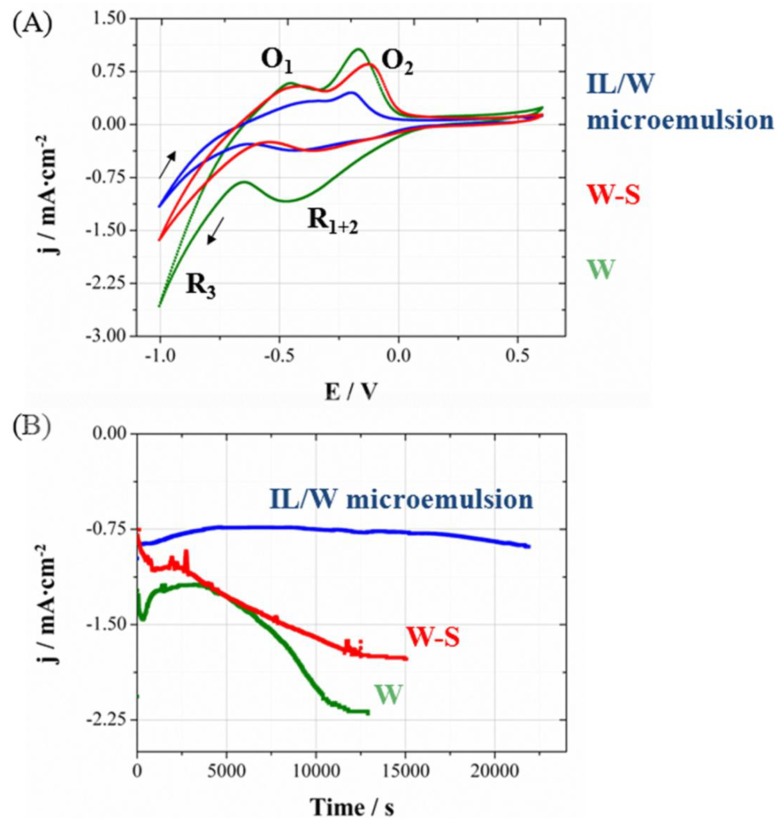
(**A**) Cyclic voltammetrie sand (**B**) chronoamperometric curves at 25 °C on Au sputtered 20 µm-thick polycarbonate membranes with 200 nm pore diameters size. Geometrical area has been used to calculate current density.

In order to determine if it is possible to apply the same potential to perform the deposition of CoPt nanowires, the processes were studied in the polycarbonate membrane, because the deposition through the membrane could be modified. The voltammetries (Figure 3A), corresponding to the deposition process in polycarbonate membranes coated with gold layer, show also a platinum reduction currentband and protons reduction over the first deposited platinum (*R*_1+2_ = *R*_1_ + *R*_2_) followed by the simultaneously CoPt deposition and hydrogen evolution (*R*_3_). The profile is similar in aqueous solution (W), aqueous solution-surfactant (79W:21S) and IL/W microemulsion systems. For the same cathodic limit previously used on Si/Ti/Au electrodes, lower proportion of the hydrogen evolution current was observed, which leads to lower O_1_ peak. Therefore, the oxidation peak of the CoPt alloy (*O*_2_) was more clearly seen. According to this analysis, CoPt nanowires were prepared potentiostatically also at −1000 mV. Figure 3B shows the chronoamperometric curves of each system under moderate stirring conditions with argon flux, trying to favor the transport of matter inside the membrane and attain a quasi-stationary regime. Electrochemical deposition from the IL/W microemulsion was significantly slower that from aqueous solution containing or not the surfactant, as a consequence of the slower transport of the electroactive species in the more viscous system.

**Figure 4:**
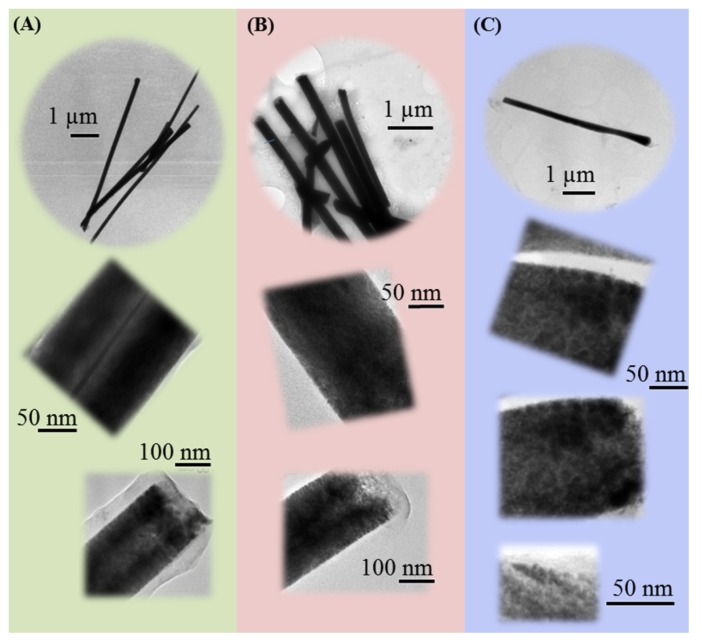
TEM micrographs of CoPt nanowires prepared in (**A**) aqueous solution (W), (**B**) aqueous solution–surfactant system (79W:21S) and (**C**) IL/W microemulsion systems at 25 °C on Au sputtered 20 µm-thick polycarbonate membranes with 200 nm pore diameters size after circulating the same charge. The first micrographs in each series correspond to a general overview of CoPt nanowires; the second one corresponds to a magnification of a central part of nanowire. In addition, the latter corresponds to a magnification of the edge of a nanowire.

Figure 4 shows the TEM micrographs of the obtained nanowires from each system (W, W-S and IL/W) after circulating the same charge. The influence of the presence of surfactant in the aqueous solution on the surface nanowire texture and the possible formation of mesoporous CoPt nanowires from the microemulsion are analyzed. In aqueous solution, larger nanowires (Table 2) than in aqueous-surfactant or microemulsion systems were obtained as corresponds to the different electrodeposition efficiencies determined from the CoPt films (Table 3). The composition of the nanowires is very similar to that of the electrodeposited films on Si/Ti/Au electrodes for each system.

**Table 2:** Elemental composition and length of CoPt nanowires obtained in different systems (W, W-S and IL/W) after circulating the same charge (6 C·cm^−2^).

System	wt.% Pt	wt.% Co	Length (µm)
W	77.5	22.5	6.9 ± 0.5
W-S	78.4	21.6	5.6 ± 0.5
IL/W microemulsion	80.2	19.8	4.9 ± 0.7

**Table 3:** Experimental circulated charge density (*q*_exp_), deposit composition, thickness (δ_exp_), calculated charge density (*q*_calc_) and efficiency of the deposition processes, at the same deposition potential (−1000 mV), for each system on Si/Ti (15 nm)/Au (100 nm) electrode.

System	*q*_exp_ (mC·cm^−2^)	wt.% Pt	wt.% Co	Thickness (nm)	*q*_calc_ (C·cm^−2^)	ε (%)
W	6.0	77.7	22.3	210	0.7	~12
W-S	6.5	79.8	20.2	150	0.5	~8
IL/W microemulsion	7.5	80.3	19.7	140	0.5	~6

The magnified TEM micrographs (Figure 4A,B) show compact nanowires obtained from both W and W-S systems, except in the extreme of the nanowires, in which the material is being incorporated. However, the CoPt nanowires obtained in IL/W microemulsion were less compact (Figure 4C). In the magnified micrographs, pores are clearly seen. The pore’s size can’t be measured in TEM images because it was very low, of a few nanometers, as corresponds to the determined droplet size in IL/W microemulsions (4.2 nm). Therefore, the electrodeposition in IL/W microemulsion allows obtaining mesoporous structures in which the small pores must correspond to the size of the droplets of the electrolytic aqueous component of the microemulsion. The IL main phase is like a template for the confined electrodeposition. The mesoporous structure of the CoPt nanowires implies higher surface area that of the compact ones, which will be corroborated if mesoporous CoPt nanowires are more catalytic to methanol oxidation than compact ones.

**Figure 5:**
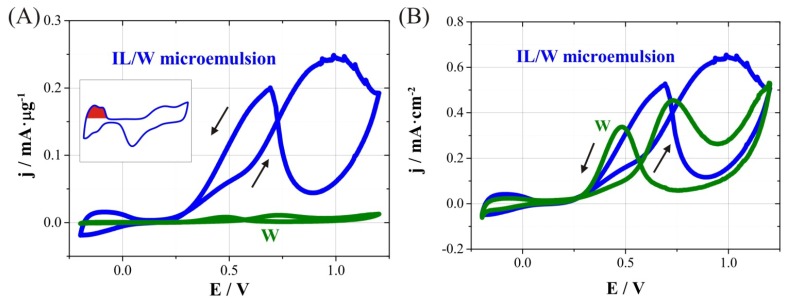
Cyclic voltammograms for methanol oxidation on CoPt nanowires obtained from W and IL/W systems. Scans were recorded in Ar saturated 1.0 M CH_3_OH/0.5 M H_2_SO_4_ at 100 mVs^−1^. Current density calculated using (**A**) catalyst’s mass, and (**B**) electrochemically active area. Inset shows the region used to calculate electrochemically active area (in red) from a cyclic voltammogram in 0.5 M H_2_SO_4_ at 100 mV s^−1^.

With these considerations in mind, the methanol oxidation reaction in oxygen-free 1.0 M CH_3_OH/0.5 M H_2_SO_4_ electrolyte was studied. On the one hand, Figure 5A shows the cyclic voltammograms recorded at 100 mV s^‑1^ considering current density per unit mass. It can be seen an outstandingly greater activity toward methanol oxidation of nanowires prepared in IL/W microemulsion systems (mesoporous CoPt nanowires) compared to those prepared in W system (compact CoPt nanowires). The current intensity per microgram of catalyst at 0.6 V is 84 µA·µg^−1^ for porous nanowires whereas for compact ones is 5.3 µA·µg^−1^. Calculated electrochemically active areas (EAA) using the hydrogen adsorption charge from the cyclic voltammogram in 0.5 M H_2_SO_4_ for both IL/W and W nanowires were 38.0 and 2.4 m^2^·g^−1^, respectively (see inset in Figure 5A). It means that these mesoporous nanowires have a surface area 16 times greater than compact ones, and comparable to that observed for commercial carbon-supported platinum nanoparticles [39]. The greater electrochemical activity of mesoporous nanowires towards methanol oxidation is due to this larger surface area for the same amount of catalyst.

On the other hand, Figure 5B shows a comparison of cyclic voltammograms with current density calculated with EAA. The methanol oxidation process starts at 0.20 V in both W and IL/W nanowires, and the activity for compact nanowires is smaller for potential values below 0.6 V (forward scan). The methanol oxidation peak appears at less positive values for W nanowires (0.72 V) than for IL/W nanowires (around 0.95 V), although the activity per surface area of catalyst at 0.72 V is similar in both of them (0.45 and 0.39 mA cm^−2^ for W and IL/W nanowires, respectively). Moreover, typical working potentials for methanol oxidation in DMFC are between 0.4 and 0.6 V. In this region, the activity of porous nanowires is slightly greater than compact ones (from 0.06 to 0.22 for W, and from 0.10 to 0.22 mA cm^−2^ for W/IL). Therefore, the use of mesoporous nanowires has some advantages with respect to compact nanowires, since it has very better electrocatalytic behaviour towards methanol oxidationand its active area is pretty larger for the same amount of catalyst. Moreover, the catalytic performance of mesoporous nanowires is comparable to that of commercial platinum nanoparticles, and further improvements may make these materials potential candidates for DMFC electrodes.

## 3. Experimental Section

The IL/W microemulsion was prepared by mixing of aqueous component (W), p-octyl polyethylene glycol phenyl ether as known as (a.k.a.) Triton X-100 (S) and 1-Butyl-3-methylimidazolium hexafluorophosphate a.k.a. bmimPF_6_ (IL) in different proportions. The mixture was stirred during 5 min under argon bubbling, leading to transparent and stable microemulsions. The aqueous solution contains 2.5 mM CoCl_2_, 1.2 mM Na_2_PtCl_6_, 0.1 M NH_4_Cl, 10 g·dm^−3^ H_3_BO_3_ at a pH adjusted to 4.5 with NaOH solutions. The viscosity, surface tension and conductivity of the selected ionic liquid-in-water microemulsion [29] have been analyzed. The surface tension is measured using Traube stalagmometer, which enables calculating the surface tension of a medium relative to water (γ). The measure of viscosity of aqueous solution and microemulsion was performed using an Ostwald viscometer. The conductivity measurements were carried out using a Crison conductimeter GLP31 (University of Barcelona, Barcelona, Spain). The conductivity cell was a model 52-92 (Crison) with Pt electrodes and a cell constant of 1 cm^−^^1^. The temperature was controlled to ±0.05 °C by a CAT temperature sensor, model 55-31 (Crison). Dynamic Light Scattering (DLS) measures of the hydrodynamic diameter of microemulsions were determined with a Malvern 4700 Instrument (IQAC-CSIC, Barcelona, Spain). The scattering angle of the light respect to the laser beam was set to 90°, in order to obtain a minimal signal of ~80 Kcounts·s^−^^1^. The measurements were made at 25 °C. The data was analyzed by the Non-Negative Least-Squares (NNLS) algorithm. The refractive index at 25 °C was determined with Optilab®, rEX.

The electrochemical experiments of CoPt deposition were performed at room temperature (25 °C) using a three-electrode electrochemical system with Si/Ti (15 nm)/Au (100 nm) substrates or polycarbonate membranes (20 µm-thick polycarbonate membranes with 200 nm pore diameters size metalized by sputtering with gold on one side), Pt spiral, and Ag/AgCl/1 M KCl as working, counter, and reference electrodes, respectively. Vacuum evaporation was used to coat the membranes with around a 100 nm-thick gold layer, enabling conductivity. Prior to the electrodeposition, the porous template was kept in the different media for 24 h to make the pores hydrophilic for uniform filling of the pores. A microcomputer-controlled potentiostat/galvanostat Autolab with PGSTAT30 equipment (University of Barcelona, Barcelona, Spain) and General Purpose Electrochemical System (GPES) software was used for the preparation of deposits.

The morphology of the deposited CoPt nanowires was examined by using Transmission Electron Microscopy (Hitachi 800 MT, CCiTUB, Barcelona, Spain) and Field Emission Scanning Electron Microscopy FE-SEM (Hitachi H-4100FE, CCiTUB, Barcelona, Spain). X-ray analyzer incorporated in Leica Stereo Scan S-360 Equipment (CCiTUB, Barcelona, Spain) was used to determine elemental composition of the deposits.

For TEM observation and the test of the CoPt nanowires as electrocatalysts for the methanol oxidation reaction, the nanowires were extracted from the polycarbonate membrane. The sputtered gold layer was dissolved with I_2_/I^−^ solution and the polycarbonate membrane was dissolved with chloroform, and washed with chloroform (x3), ethanol (x2) and water (x2). Polycarbonate membranes have been selected in order to reduce the possible oxidation of CoPt alloy and the cost of the methodology due to the smoother nanowires release treatments (dissolving in organic solvents) than the used in alumina membranes, and the much lower cost. To test the behavior of the synthesized nanowires, a glassy carbon (GC) electrode (0.071 cm^2^) was used as substrate for the catalyst (working electrode). Previous to each test, the GC electrode was polished with alumina 0.05 µm to obtain a mirror finish, and it was rinsed with Milli Q water in an ultrasonic bath. Nanowires were deposited onto the GC electrode by means of ink composed of 5 mg of nanowires, 525 µL of water, 175 µL of ethanol, and 88.5 µL of 5 wt.% Nafion solution. Five microliters of the ink were dropped onto the electrode and dried at room temperature resulting in a homogenous coating. This leads to a final catalyst loading of 31.7 µg. In order to clean and activate the electrode surface and to study the methanol electrooxidation process, the electrolyte was purged with argon for 30 min to deareate the system, and after the samples were cycled at 100 mV s^−1^ between −0.21 and 1.2 V until reproducible voltammograms were obtained. Methanol electrooxidation was studied by cyclic voltammetry, recorded at 100 mV s^−1^, in a Ar saturated 1.0 M CH_3_OH/0.5 M H_2_SO_4_solution.

The electrochemically active surface area of each catalyst was calculated using the hydrogen adsorption charge from the cyclic voltammogram in 0.5 M H_2_SO_4_ at 100 mV s^−1^. The charge density associated with a monolayer of hydrogen atoms adsorbed on polycrystalline platinum (210 µC cm^−2^) was assumed. The active surface area was calculated by integration of the area under hydrogen adsorption region and subtracting the double layer contribution.

All solutions were prepared with doubly distilled water treated with a Millipore Milli-Q system. In the manuscript, all potentials are referred to the Ag/AgCl/1 M KCl electrode.

## 4. Conclusions

The use of microemulsions with confined nanometric droplets of ionic liquid dispersed in a continuous CoPt electrolytic solution, in the presence of surfactant, has allowed us to grow electrolytically mesoporous CoPt nanowires, several microns long, in the channels of gold-coated polycarbonate membranes. The insolubility of the ionic liquid in the electrolytic solution causes that the electrodeposition occurs only from the aqueous component of the microemulsion. The new electrochemical method proposed opens the possibility of easy synthesis of different mesoporous micro/nanometric materials (metals or alloys), useful for catalytic applications, without need of employing aggressive reducing agents. The size of the droplets in the microemulsion defines the pore size of the synthesized mesoporous material.

The synthesized mesoporous CoPt nanowires present a much enhanced catalytic behavior for methanol oxidation reaction in acidic medium, respect to that observed for compact CoPt nanowires of the same composition, and comparable to that observed for commercial carbon-supported platinum nanoparticles. This fact corroborates the significantly greater surface area of the mesoporous nanowires. These nanowires can be a potential alternative for methanol fuel cells, because they promote the catalytic activity and reduce costs respect to pure-platinum nanoparticles.
